# Greater Accuracy of the eGFR Formula by Using a Power Function of Patient Height over Its Unidimensional Value in Pediatric Kidney Transplantation

**DOI:** 10.3390/jcm15072512

**Published:** 2026-03-25

**Authors:** Jeffrey J. Gaynor, Mahmoud Morsi, Jayanthi Chandar, Marissa Defreitas, Angel Alvarez, Matthew Gaynor, Junichiro Sageshima, Gaetano Ciancio

**Affiliations:** 1 Department of Surgery, University of Miami Miller School of Medicine, Jackson Memorial Hospital, Miami, FL 33136, USAgciancio@med.miami.edu (G.C.); 2 Miami Transplant Institute, University of Miami Miller School of Medicine, Jackson Memorial Hospital, Miami, FL 33136, USA; 3 Department of Pediatrics, Division of Pediatric Nephrology, University of Miami Miller School of Medicine, Jackson Memorial Hospital, Miami, FL 33136, USA; 4 Department of Surgery, University of California Davis School of Medicine, Sacramento, CA 95817, USA; 5 Department of Urology, University of Miami Miller School of Medicine, Jackson Memorial Hospital, Miami, FL 33136, USA

**Keywords:** pediatric kidney transplantation, estimated glomerular filtration rate, updated Schwartz bedside formula, patient height, serum creatinine, statistically more accurate formula

## Abstract

**Background**: The updated Schwartz and CKiDU25 bedside (SCr-based) formulae for the estimated glomerular filtration rate (eGFR) in children are defined by a constant term (with the latter formula dependent upon age and sex) multiplied by the ratio of patient’s height (m) to SCr (mg/dL). However, the Schwartz formula can severely underestimate the measured GFR (mGFR) at higher mGFR levels. **Methods**: For a single-center cohort of 92 pediatric kidney transplant recipients, we statistically determined if the log{eGFR} at 1 mo and 6 mo post-transplant might further depend upon patient demographics or height, indicating the inadequacy of these formulae for properly predicting the mGFR. We also determined how the log{SCr} at 1 mo and 6 mo post-transplant might depend upon patient demographics and height, helping to corroborate any arrived-at improved functional form for the eGFR. **Results**: Overall, our cohort received good-quality donor kidneys; however, both eGFR formulae calculated that the percentage of recipients with an eGFR < 60 mL/min/1.73 m^2^ at 1 mo and 6 mo post-transplant was 26–28%. Furthermore, neither the updated Schwartz nor the CKiDU25 bedside formulae adequately controlled for the influence of patient height on SCr; in fact, the patient height squared was superior to its unidimensional value at accounting for the sharp increase in SCr that normally occurs as children grow from infancy to young adulthood (*p* < 0.000001 at mo1, *p* = 0.000003 at mo6 for the updated Schwartz bedside formula; *p* = 0.0009 at mo1, *p* = 0.005 at mo6 for the CKiDU25 bedside formula). The log{SCr} was also best fitted by a linear regression model that controlled for the log{patient height squared} (*p* < 0.000001 at both mo1 and mo6). **Conclusions**: A statistically more accurate eGFR formula should be based on using a power function (power > 1) for patient height rather than its unidimensional value.

## 1. Introduction

For many years, the original Schwartz bedside (creatinine-based) formula was considered the gold standard for estimating the glomerular filtration rate (eGFR) in pediatric patients. This formula, introduced in 1976, was based on an analysis of 186 pediatric patients and was defined as an overall constant term multiplied by a simple ratio of body length (height in cm) divided by plasma creatinine (mg/dL) [1]. This popular formula was then revised in 1987 to include distinct constant terms depending on the pediatric patient’s age and sex [2]. The original 1976 formula was then updated in 2009 after an analysis of 349 pediatric patients defined at baseline with chronic kidney disease (CKD), i.e., the initial Chronic Kidney Disease in Children (CKiD) cohort, and was based on using serum creatinine (SCr) rather than plasma creatinine values [3]. The 2009 updated bedside formula included a simple change in the numeric value of the original single constant term. The CKiD cohort was then expanded to include more young children (<5 years of age) and young adults (ages 18–25 years), and the recently published CKiD U25 bedside formula, based on an analysis of 2655 observations of 928 participants, included the same original ratio of patient height (m) to serum creatinine (mg/dL) but was multiplied by a statistically more complicated constant term that depended on the pediatric patient’s age and sex, using separate sex-specific log linear terms for ages 1–12 years and 12–18 years, i.e., two knots, and sex-specific constant terms for ages 18–25 years [4].

While each of these reports [1,2,3,4] provided statistical justification of their arrived-at formulations by including goodness-of-fit comparisons with the measured GFR (mGFR), some studies have reported a lack of linear fit of the {patient height to serum (or plasma) creatinine concentration} ratio vs. mGFR [5,6,7,8,9,10]. Specifically, the eGFR as determined by the original (or updated) Schwartz bedside formula appeared to severely underestimate the mGFR at higher mGFR levels. In fact, in a single-center, retrospective review of 155 pediatric kidney transplant recipients, Siddique et al. [8] found that the mean mGFR starting at 6 mo post-transplant was 25.81% higher in comparison with the mean eGFR as determined by the updated Schwartz bedside formula. Exactly how well the more recently derived CKiD U25 bedside formula improves the goodness-of-fit at higher mGFR levels in pediatric kidney transplant recipients post-transplant is not clear.

It is known that there is a huge increase in lean body mass, muscle volume, extracellular volume, and total body water (i.e., modern physiology-based compartments) as healthy children age from infancy to young adulthood, which concomitantly reflects a huge increase in SCr that occurs as healthy children age from 2 to 19 years. The ratio of highest-to-lowest median SCr as children age annually from 2 to 19 years is roughly 2.2 among females and 2.7 among males [11,12,13]. Interestingly, while patient height has been conventionally used as an indirect proxy for these compartments, the ratio of highest-to-lowest median height as children age annually from 2 to 19 years is less, being 1.9 among females and 2.1 among males [14]. These numbers therefore suggest that a power function of patient height > 1.0 would more accurately reflect the increase in SCr that normally occurs as children grow from infancy to adulthood, rather than the more conventionally used unidimensional value of height.

For a cohort of 92 pediatric kidney transplant recipients who were recently transplanted at our center, one goal was to determine if there was any evidence of renal function being underestimated by the updated Schwartz or CKiD U25 bedside formula for the eGFR [3,4] early post-transplant, i.e., at 1 mo and 6 mo post-transplant, prior to any major influence of other clinical events, such as biopsy-proven acute rejection (BPAR). Furthermore, we wanted to statistically determine if either of these eGFR formulae might further depend upon any patient demographic variables (i.e., age, sex, or race/ethnicity) or body length. Any residual dependency of these eGFR formulae on patient demographics or body length would imply that they were statistically inadequate, and we were particularly interested in determining whether the eGFR was satisfactorily modeled by the unidimensional value of patient height (vs. greater accuracy being provided by using a power function for patient height). Similarly, we also wanted to determine exactly how patients’ SCr at 1 mo and 6 mo post-transplant might depend upon patient demographics and patient height. Clearly, a linear model that accurately depicts SCr as a function of these characteristics might also influence how to optimally formulate the eGFR. The results of this observational study are presented here.

## 2. Patients and Methods

Between January 2015 and December 2023, 92 consecutively transplanted pediatric recipients (age < 19 yr) of either deceased donor (DD) or non-HLA identical living donor (LD) kidney-alone transplants were included in this study. The pediatric recipient inclusionary criteria included being scheduled to receive an ABO-compatible, kidney-alone transplant. The pediatric recipient exclusionary criteria included the following: patients who had a current malignancy or history of malignancy (within the past 5 years), except for localized basal or squamous cell carcinoma of the skin that was successfully treated; patients who had significant liver disease, uncontrolled concomitant infections and/or severe diarrhea, vomiting, active upper gastro-intestinal tract malabsorption; or patients with a screening/baseline total white blood or platelet count suggestive of leukopenia or thrombocytopenia.

These 92 kidney transplants were performed by two of our transplant surgeons using surgical modifications (as previously reported) to the conventional kidney transplant technique [15,16]. When following these surgical modifications, no initial ureteral stent placement or surgical drainage was used. This study was approved by the Institutional Review Board at the University of Miami (IRB #20140129) (most recent renewal date: 24 October 2025) and followed the ethical principles (as revised in 2013) of the Helsinki Declaration. Study participant consent was waived due to the retrospective nature of the study.

All patients were scheduled to receive dual-agent induction with rabbit anti-thymocyte globulin (Thymoglobulin^®^) (either 1 or 3 doses at 1.0 mg/kg/dose, with the first dose being administered intra-operatively and the other 2 doses, if scheduled, being administered during days 2–4 post-transplant) and basiliximab (Simulect^®^) (2 doses, with the first dose being administered intra-operatively and the second dose scheduled to be given at day 3–4 post-transplant). Patients with a pretransplant weight < 35 kg were scheduled to receive two 10 mg basiliximab doses, and patients with a pretransplant weight ≥ 35 kg were scheduled to receive two 20 mg basiliximab doses. Additionally, the induction protocol specified that patients who were either crossmatch positive, highly sensitized, or had specific causes of end-stage renal disease (e.g., primary focal segmental glomerulosclerosis) were eligible to receive up to one 375 mg/m^2^ dose of Rituximab as part of induction.

Oral tacrolimus was scheduled to be introduced when the serum creatinine was ≤3 mg/dL. Tacrolimus was initiated at 0.1 mg/kg twice daily after renal function had improved, with a target (12 h) trough level of 4–8 ng/mL. The target enteric-coated mycophenolate sodium (EC-MPS) dosing was 600 mg/m^2^ twice daily introduced on the second postoperative day. Dose adjustment was performed according to the white blood cell count and gastrointestinal tolerance. Any withholding of EC-MPS for a minimum period of one month was documented along with the reasons for withholding. Methylprednisolone was given intravenously at 10 mg/kg/day (maximum 500 mg/day) for three days postoperatively followed by scheduled early withdrawal during the first postoperative week or shortly after hospital discharge (i.e., corticosteroid avoidance).

Scheduling of non-immunosuppressive adjunctive therapy was essentially the same as in our previously described protocols [17,18]. For example, regarding cytomegalovirus (CMV) prophylaxis, all patients were treated immediately post-transplant with intravenous ganciclovir for 3 d, followed by daily valganciclovir orally for 3 mo, with the doses based on renal function. In donor CMV Ig+/recipient CMV Ig- combinations, treatment was given for 6 mo postoperatively. In patients developing rejection that required steroids or antilymphocyte therapy, intravenous ganciclovir or valganciclovir was reinstituted. Pneumocystis prophylaxis with trimethoprim–sulfamethoxazole was also given for a minimum of 12 mo [17,18].

Of note, all the DD organs were immediately placed at the time of arrival at our center in a LifePort renal preservation machine with Kidney Perfusion Solution (KPS-1) [19].

Delayed graft function (DGF) was defined as the requirement for dialysis during the first week post-transplant. All patients were followed for the incidence of BPAR, any viral viremia (CMV, BK, EBV, etc.), surgical complications, and infections that required hospitalization. The Banff criteria were used to determine rejection severity [20], with BPAR requiring both clinical indication and treatment of the episode. Cases of biopsy-proven antibody-mediated rejection (AMR) were also determined [21], along with documentation of the incidence of de novo DSA+. Renal function was determined using both the updated Schwartz bedside formula for the eGFR and the more recent CKiD U25 bedside eGFR formula (SCr-based formulae) [3,4]. Graft loss was determined as the time of re-establishment of long-term dialysis (death-censored graft failure, DCGF) or death (i.e., death with a functioning graft, DWFG).

### Statistical Analysis

The means and standard errors (SEs) were determined for all continuous (baseline and outcome) variables, along with their medians and ranges of values. For the categorical variables, percentages of patients having the selected characteristics were determined. In an attempt to avoid any major influence of non-relevant clinical events, e.g., BPAR occurrence, the statistical analysis in this study was limited to the clinical outcomes that occurred during the first 6 mo post-transplant.

The renal function at 1 mo and 6 mo post-transplant was determined using 2 distinct eGFR determinations: one by the updated Schwartz bedside formula and one by the more recently developed CKiD U25 bedside formula [3,4]. Specifically, the updated Schwartz bedside formula is defined as 41.3*height (m)/SCr (mg/dL), and the CKiD U25 bedside formula is defined as k* height (m)/SCr (mg/dL), where the multiplying constant k is defined as follows: for females, it equals 36.1*(1.008**(age-12)) for 1 ≤ age < 12 years, 36.1*(1.023**(age-12)) for 12 ≤ age < 18 years, and 41.4 for age ≥ 18 years; and for males, it equals 39.0*(1.0008**(age-12)) for 1 ≤ age < 12 years, 39.0*(1.045**(age-12)) for 12 ≤ age < 18 years, and 50.8 for age ≥ 18 years.

Since both eGFR formulae were originally derived on the natural logarithm scale [3,4], we followed a similar approach here by using log-transformed eGFR values. Thus, a stepwise linear regression of log {eGFR} was performed to statistically determine whether the eGFR (as defined above) might further depend upon: the patient age at transplant (considered as a continuous variable as well as a dichotomous variable comparing age < 13 vs. ≥13 years, the median age at transplant), sex, race/ethnicity (considering non-Hispanic Black and Hispanic as 2 distinct dichotomous variables), and patient height (cm) (considered as a continuous variable on its natural logarithmic scale). A strict type I error of 0.01 was used in the attempt to avoid any spurious associations. Additionally, no imputation for the eGFR was performed in patients who experienced death-censored graft failure prior to 6 mo post-transplant.

It is well known that biochemical determinations have skewed distributions and that determinations such as SCr are best modeled using natural logarithmic transformed values, i.e., are more appropriately fitted by log-normal (rather than normal) distributions [22,23,24,25]. Thus, we similarly performed a stepwise linear regression of log {SCr} to determine its significant multivariable predictors, considering patient age, sex, race/ethnicity, and height (considered as a continuous variable on its natural logarithmic scale) using a strict type I error of 0.01 in the attempt to avoid any spurious associations.

## 3. Results

### 3.1. Overall Results

The distributions of selected baseline variables appear in Table 1. The median recipient age was 13.0 yr (range: 2.3–18.9 yr), and males comprised 64.1% (59/92). Non-Hispanic Blacks and Hispanics comprised most of the patients, 33.7% (31/92) and 43.5% (40/92), respectively. The percentages of patients who received primary, pre-emptive, and LD kidney transplants were 96.7% (89/92), 28.3% (26/92), and 35.9% (33/92), respectively. The median Kidney Donor Profile Index (KDPI) among the 59 DD recipients was 12% (range: 1–61%). The median donor age was 29.5 yr (range: 2–60 yr), and 13.0% (12/92) had pre-existing DSAs.

The distributions of selected outcome variables appear in Table 2. In terms of overall results, the incidence of DGF was 1.1% (1/92), and 8.7% (8/92) developed a first BPAR during the first 6 mo post-transplant. One patient experienced (death-censored) graft failure during the first 6 mo post-transplant due to renal vein thrombosis (N = 1, at day 1 post-transplant). None of the patients experienced death with a functioning graft during the first 6 mo post-transplant.

Table 2 shows that the median recipient height at 1 mo and 6 mo post-transplant was 143.0 [range: 81.0–181.0] and 145.0 [range: 82.0–181.0] cm, respectively. The median SCr at 1 mo and 6 mo post-transplant was 0.70 [range: 0.24–3.56] and 0.77 [range: 0.22–3.30] mg/dL, respectively. Using the updated Schwartz bedside formula, the median eGFR at 1 mo and 6 mo post-transplant was 80.3 [range: 18.9–160.9] and 75.9 [range: 19.7–153.9] mL/min/1.73 m^2^, respectively. Using the CKiD U25 bedside formula, the median eGFR at 1 mo and 6 mo post-transplant was 76.8 [range: 19.7–145.2] and 73.1 [range: 18.3–152.3] mL/min/1.73 m^2^, respectively.

Lastly, Table 2 shows that according to the updated Schwartz bedside formula, the percentage of patients with an eGFR < 60 mL/min/1.73 m^2^ was 27.5% (25/91) at both 1 mo and 6 mo post-transplant. According to the CKiD U25 bedside formula, the percentage of patients with an eGFR < 60 mL/min/1.73 m^2^ was 26.4% (24/91) and 27.5% (25/91) at 1 mo and 6 mo post-transplant, respectively.

### 3.2. Stepwise Linear Regression (Multivariable) Analyses of Log {eGFR} at 1 Mo and 6 Mo Post-Transplant

**Figure 1:**
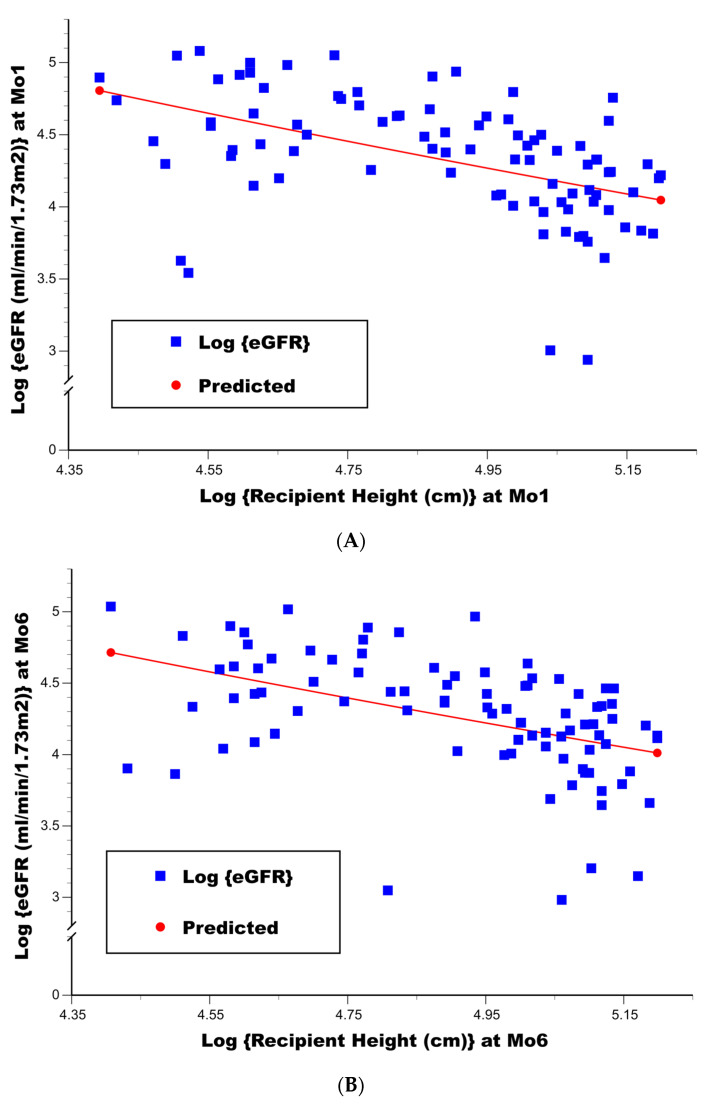
(**A**) Scatterplot of log {recipient height (cm)} vs. log {eGFR (mL/min/1.73 m^2^)} (using Schwartz’s updated bedside formula) at 1 mo post-transplant, along with the linear regression (least squares) prediction line (N = 91). (**B**) Scatterplot of log {recipient height (cm)} vs. log {eGFR (mL/min/1.73 m^2^)} (using Schwartz’s updated bedside formula) at 6 mo post-transplant, along with the linear regression (least squares) prediction line (N = 91).

In analyzing the updated Schwartz bedside formula, the stepwise linear regression of log {eGFR} at both 1 mo and 6 mo post-transplant found a significant inverse association of higher log {recipient height (cm)} with a lower log {eGFR} (*p* < 0.000001 at 1 mo in Table 3; *p* = 0.000003 at 6 mo in Table 4). Scatterplots of log {recipient height (cm)} vs. log {eGFR} at 1 mo and 6 mo appear in Figure 1A,B, respectively, along with overlays of the predicted regression lines. Once this single variable was selected into the linear regression model for the log {eGFR}, no other variables contained additional predictive value (*p* > 0.15 in Table 3; *p* > 0.20 in Table 4). The coefficient of multiple determination, R^2^, achieved for each linear model was 0.25 and 0.22, respectively. Of note, the model coefficient ± SE for the log {recipient height (cm)} was −0.944 ± 0.173 in Table 3 and −0.887 ± 0.178 in Table 4 (95% confidence intervals for these model coefficients are also included in the tables), suggesting that at both 1 mo and 6 mo post-transplant the eGFR would statistically be more accurately represented by using the square of recipient height (cm), i.e., constant*(height**2)/Scr, rather than its unidimensional value in the updated Schwartz bedside formula.

In analyzing the CKiD U25 bedside formula, even though the constant term developed in this formula accounts for differences in the eGFR by patient age and sex, the stepwise linear regression of log {eGFR} at both 1 mo and 6 mo post-transplant still found a significant inverse association of a higher log {recipient height (cm)} with a lower eGFR (*p* = 0.0009 at 1 mo in Table 5; *p* = 0.005 at 6 mo in Table 6). Once this single variable was selected into the linear regression model for the log {eGFR}, no other variables contained additional predictive value (*p* > 0.10 in Table 5; *p* > 0.40 in Table 6). The coefficient of multiple determination, R^2^, achieved for each linear model was 0.12 and 0.09, respectively. Thus, while the statistical association of the log {recipient height (cm)} with the log {eGFR} was less strong when using the CKiD U25 bedside formula instead of the updated Schwartz bedside formula, the recipient height effect was nonetheless statistically significant, implying that even the CKiD U25 bedside formula does not adequately account for differences in the measured GFR according to recipient height.

### 3.3. Stepwise Linear Regression (Multivariable) Analyses of Log {SCr} at 1 Mo and 6 Mo Post-Transplant

**Figure 2:**
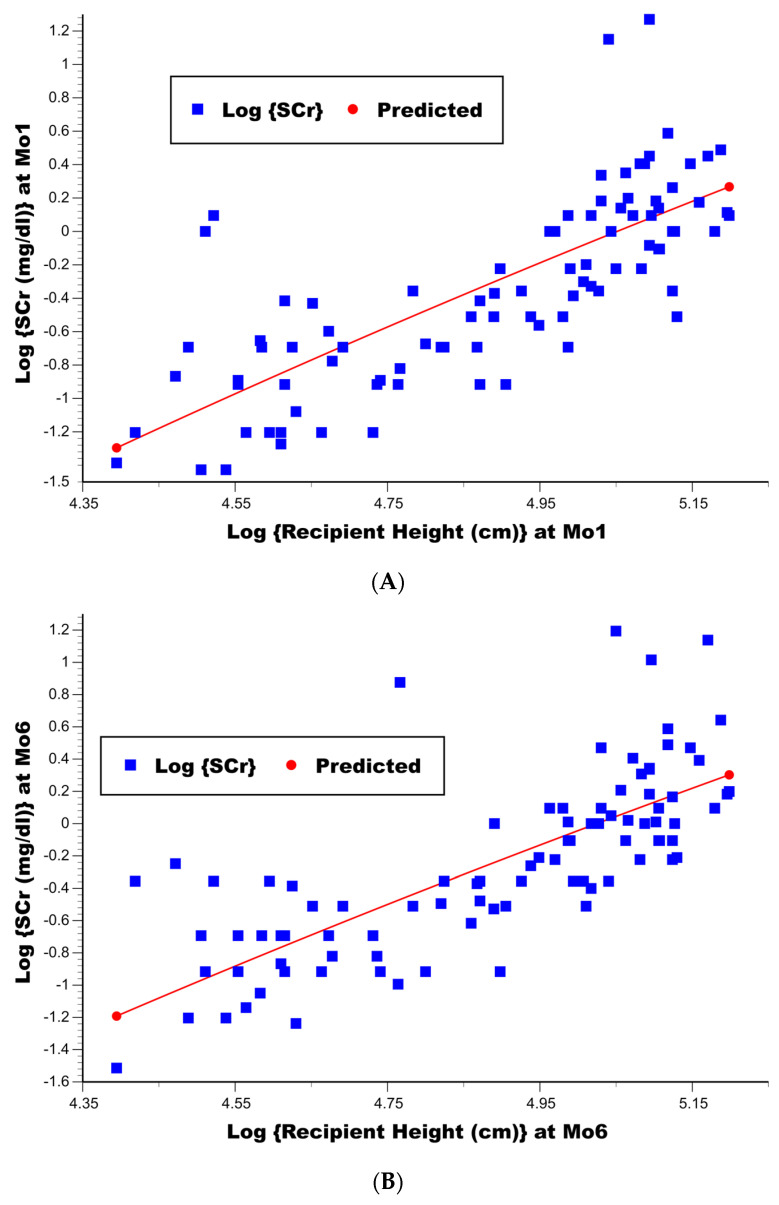
(**A**) Scatterplot of log {recipient height (cm)} vs. log {SCr (mg/dL)} at 1 mo post-transplant, along with the linear regression (least squares) prediction line (N = 91). (**B**) Scatterplot of log {recipient height (cm)} vs. log {SCr (mg/dL)} at 6 mo post-transplant, along with the linear regression (least squares) prediction line (N = 91).

The stepwise linear regression of log {SCr} at both 1 mo and 6 mo post-transplant found a significant positive association of a higher log {recipient height (cm)} with a significantly higher log {SCr} (*p* < 0.000001 at 1 mo in Table 7; *p* < 0.000001 at 6 mo in Table 8). Scatterplots of log {recipient height (cm)} vs. log {SCr} at 1 mo and 6 mo appear in Figure 2A,B, respectively, along with overlays of the predicted regression lines. Once this single variable was selected into the linear regression model for the log {SCr}, no other variables contained additional predictive value (*p* > 0.15 in Table 7; *p* > 0.20 in Table 8). The coefficient of multiple determination, R^2^, achieved for each linear model was 0.59 and 0.56, respectively. Of note, the model coefficient ± SE for the log {recipient height (cm)} was 1.944 ± 0.173 in Table 7 and 1.887 ± 0.178 in Table 8 (95% confidence intervals for these model coefficients are also included in the tables), suggesting that at both 1 mo and 6 mo post-transplant, the optimal adjustment for differences in the log {SCr} by recipient height would statistically be best achieved by using the recipient height (cm) squared rather than its unidimensional value. In fact, the F-test of H_0_ when the model coefficient for log {recipient height (cm)} equals 2.0 yields *p* = 0.75 in Table 7 and *p* = 0.53 in Table 8; conversely, the F-test of H_0_ when the model coefficient for log {recipient height (cm)} equals 1.0 yields *p* < 0.000001 in Table 7 and *p* = 0.000003 in Table 8.

### 3.4. An Attempt at Replacing the Recipient Height by Its Squared Value in the eGFR Bedside Formula

The stepwise linear regression results for the log {eGFR} based on the updated Schwartz bedside formula suggest that replacing its unidimensional height value with the recipient height (m) squared in the formula may more accurately depict the mGFR among pediatric kidney transplant recipients during the first 6 mo post-transplant. This suggestion is also supported by our stepwise linear regression results for the log {SCr} during the first 6 mo post-transplant. Thus, without having the actual mGFR values, if we utilize the Siddique et al. [8] finding that the mean mGFR at roughly 6 mo post-transplant is 25.81% higher compared with the mean eGFR determined using the updated Schwartz bedside formula, then our overall mean eGFR at 6 mo post-transplant (shown in Table 2) would increase to 97.4 (i.e., 77.4 × 1.2581) mL/min/1.73 m^2^. Since our mean “Recipient Height (m) squared/SCr at 6 mo post-transplant” equals 2.4801, dividing 97.4 by 2.4801 would yield the constant K = 39.28. An additional estimate of K as 36.84 would similarly be obtained if one assumed that the mean mGFR was only 18% higher than the mean eGFR determined from the updated Schwartz bedside eGFR formula.

Using K = 39.28 in the formula eGFR = K*recipient height (m) squared/SCr (mg/dL), the mean eGFR ± SE at 1 mo and 6 mo would increase to 102.7 ± 3.4 [N = 91; median = 101.4; range: 29.3–187.0] and 97.4 ± 3.2 [N = 91; median = 98.5; range: 24.6–189.7], respectively, and the percentages of patients having an eGFR < 60 mL/min/1.73 m^2^ at 1 mo and 6 mo would decline to 4.4% (4/91) and 9.9% (9/91), respectively, which are much more in line with our expectations. If K = 36.84 is assumed, then the mean eGFR ± SE at 1 mo and 6 mo would increase to 96.3 ± 3.2 [N = 91; median = 95.1; range: 27.5–175.4] and 91.4 ± 3.0 [N = 91; median = 92.4; range: 23.0–177.9], respectively, and the percentages of patients having an eGFR < 60 mL/min/1.73 m^2^ at 1 mo and 6 mo would become 8.8% (8/91) and 12.1% (11/91), respectively, still less than half of the percentages estimated by the updated Schwartz bedside and CKiD U25 bedside formulae (see Table 2).

### 3.5. A Simple Explanation for the Underestimation of the True GFR by the Updated Schwartz and CKiD U25 Bedside Formulae

In our cohort, the ratio between the 75th and 25th percentiles for SCr at both mo1 and mo6 post-transplant is 1.10/0.50 = 2.20. Similarly, the ratio between the 95th and 5th percentiles for SCr at mo1 and mo6 post-transplant is 1.63/0.30 = 5.43 and 1.90/0.32 = 5.94, respectively. An optimal representation of recipient height in an eGFR formula should be able to account for most of the huge variation taking place in these SCr values. As can be seen in Table 2, the ratio of largest-to-smallest recipient height at mo1 and mo6 post-transplant is 1.81/0.81 = 2.23 and 1.81/0.82 = 2.21, respectively. The square of these ratios yields 4.99 and 4.87, respectively. Clearly, the use of unidimensional recipient height accounts for less than 50% of the full range of SCr values, whereas the square of recipient height accounts for 80–90% of the full range of SCr values. The ratio of the constant k term in the CKiD U25 bedside formula for the eGFR from age 19 to 2 years (the age range in our cohort) yields 50.8/36.0 = 1.41 for males and 41.4/33.3 = 1.24 for females. Clearly, even multiplying one of these ratios by the recipient height would still not account for the full range of recipient SCr values as that offered by using the recipient height squared.

## 4. Discussion

We believe that this single-center, observational study of 92 pediatric kidney-alone transplant recipients provides the following important clinical results pertaining to renal function during the early post-transplant period (i.e., at 1 mo and 6 mo): (i) Neither the updated Schwartz nor the CKiD U25 bedside eGFR formulae [3,4] appear to adequately control for the influence of patient height on SCr; in fact, our data suggest that the patient height squared would be superior to using its unidimensional value in attempting to account for the huge increase normally observed in SCr as children grow from infancy to young adulthood. (ii) Our linear regression results for the eGFR are similarly reflected in the linear regression results obtained for the recipients’ SCr, which also suggest that the log {SCr} is best fitted by a simple linear regression model of log {patient height squared}. iii) Both the updated Schwartz and CKiD U25 bedside eGFR formulae appear to severely underestimate the mGFR in some cases during the early post-transplant period; clearly, among a cohort of pediatric kidney transplant recipients who overall received good-quality donor kidneys, the percentages of recipients with an eGFR < 60 mL/min/1.73 m^2^ should not be approaching 26–28% at 1 mo and 6 mo post-transplant.

While we are not trying to claim that the mGFR is better fitted across all types of pediatric patients by using a ratio of patient height squared to SCr, we do believe that a power function of patient height (with power > 1) would likely yield more accurate results compared with simply using its unidimensional value. Other investigators could consider using this approach in the future. Recently, investigators at the European Kidney Function Consortium (EKFC) have introduced an eGFR formula that is based on the ratio of a parameter Q to SCr, where Q is defined as a fourth-degree polynomial function of either patient age or patient height. Clearly, the parameter Q was designed to more optimally account for the huge increase that normally occurs in SCr as children grow from infancy to adulthood [26,27,28,29,30].

In Table 7 and Table 8, the regression coefficients for the log {recipient height (cm)} are approximately 1.9 at both time points (i.e., at 1 mo and 6 mo post-transplant). This key result supports the idea of a quadratic relationship existing between SCr and height (at least based on our data), as the fitted linear model, Log{SCr} = α + βLog{height}, is reasonably approximated by Log{SCr} = α + 2Log{height}, implying that Log{SCr}-2Log{height} = Log {SCr/height^2^} is equal to a simple constant, α. This result, while not in the exact shape, supports the approach taken by the EKFC [26,27,28,29,30].

Finally, the current study suffers from numerous important study limitations. First and foremost, we had no mGFR values with which to compare the eGFR in our study. Second, while a single-center pediatric kidney transplant sample size of 92 patients is reasonably large, we did not have access to another cohort of non-kidney transplanted pediatric patients. Third, as this was a single-center observational study, validation would still require the reporting of similar results by other centers prior to generalizing these study results (in some way) to the overall pediatric kidney transplant population. Fourth, we did not consider analyzing other representations of body size/muscle mass in this study, such as a power function of body weight or power functions of both patient height and body weight (similar to those used in the conventional definitions of body surface area). Lastly, modeling the eGFR in pediatric kidney transplant recipients at 12 mo post-transplant or beyond would be a more complex endeavor, as renal function at those times would certainly depend upon the prior occurrence(s) of clinical events such as BPAR, viral infections that required hospitalization, and chronic allograft injury.

## Figures and Tables

**Table 1:** Distributions of selected baseline variables (N = 92).

Mean ± SE If Continuous [Median and Range also Included]	
Baseline Variables	Percentage with Characteristic If Categorical
DOT	
<2020	43.5% (40/92)
≥2020	56.5% (52/92)
Recipient Age (yr)	11.7 ± 0.6 (N = 92)
	[Median = 13.0; Range: 2.3–18.9]
Recipient Gender	
Female	35.9% (33/92)
Male	64.1% (59/92)
Recipient Race/Ethnicity	
Black (non-Hispanic)	33.7% (31/92)
Hispanic	43.5% (40/92)
White (non-Hispanic)	18.5% (17/92)
Other ^1^	4.3% (4/92)
Recipient Height (cm)	134.6 ± 3.0 (N = 92)
	[Median = 140.4; Range: 77.0–180.1]
Recipient Weight (kg)	38.2 ± 2.2 (N = 92)
	[Median = 34.9; Range: 11.9–98.5]
Recipient BMI (kg/m^2^)	19.4 ± 0.5 (N = 92)
	[Median = 18.2; Range: 11.8–36.2]
Retransplant Status	
Primary	96.7% (89/92)
Retransplant	3.3% (3/92)
Pre-emptive Transplant	
No	71.7% (66/92)
Yes	28.3% (26/92)
Pretransplant Time on Dialysis (mo)	19.8 ± 2.3 (N = 92)
[Note: Scored 0 if Pre-emptive]	[Median = 14.9; Range: 0.0–98.9]
Cause of ESRD	
Acquired	38.0% (35/92)
Congenital	57.6% (53/92)
Unknown	4.3% (4/92)
Donor Type	
Living	35.9% (33/92)
Deceased	64.1% (59/92)
Donor Age (yr)	29.8 ± 1.2 (N = 92)
	[Median = 29.5; Range: 2.0–60.0]
CIT (h)	15.6 ± 1.3 (N = 92)
	[Median = 19.9; Range: 0.4–48.6]
CIT (h) among LD Recipients	1.0 ± 0.1 (N = 33)
	[Median = 0.9; Range: 0.4–2.9]
CIT (h) among DD Recipients	23.8 ± 0.9 (N = 59)
	[Median = 22.6; Range: 5.6–48.6]
KDPI (%)	15.2 ± 1.7 (N = 59)
	[Median = 12; Range: 1–61]
KDPI (%)	
<20	66.1% (39/59)
20–34	28.8% (17/59)
≥35 ^2^	5.1% (3/59)
Pretransplant cPRA (%)	6.5 ± 1.9 (N = 92)
	[Median = 0; Range: 0–98]
Pretransplant cPRA (%)	
0	77.2% (71/92)
1–19	12.0% (11/92)
≥20	10.9% (10/92)
Pre-existing DSA+	
No	87.0% (80/92)
Yes	13.0% (12/92)

^1^ Other race/ethnicity included Asian or Middle Eastern (4/92). ^2^ For 3 recipients of deceased donor kidneys with a KDPI ≥ 35%, the actual KDPI was 48%, 56%, and 61%, respectively.

**Table 2:** Distributions of selected outcome variables (N = 92).

Mean ± SE if Continuous [Median and Range also Included]	
Outcome Variables	Percentage with Characteristic If Categorical
**Overall results during 1st 6 mo post-transplant**	
Developed DGF	
No	98.9% (91/92)
Yes	1.1% (1/92)
Developed a 1st BPAR ^1^	
No	91.3% (84/92)
Yes	8.7% (8/92)
Developed (Death-Censored) Graft Failure ^2^	
No	98.9% (91/92)
Yes	1.1% (1/92)
Death with a Functioning Graft ^2^	
No	100.0% (92/92)
Yes	0.0% (0/92)
Developed (Death-Uncensored) Graft Loss ^2^	
No	98.9% (91/92)
Yes	1.1% (1/92)
**Renal Function at 1 mo Post-transplant**	
Recipient Height (cm)	135.8 ± 3.0 (N = 91)
	[Median = 143.0; Range: 81.0–181.0]
Serum Creatinine (mg/dL)	0.84 ± 0.06 (N = 91)
	[Median = 0.70; Range: 0.24–3.56]
Updated Schwartz Bedside eGFR (mL/min/1.73 m^2^) ^3^	83.3 ± 3.4 (N = 91)
	[Median=80.3; Range: 18.9–160.9]
CKiD U25 Bedside eGFR (mL/min/1.73 m^2^) ^4^	79.3 ± 2.9 (N = 91)
	[Median = 76.8; Range: 19.7–145.2]
Percentage with an Updated Schwartz Bedside eGFR	
<60 mL/min/1.73 m^2^	27.5% (25/91)
Percentage with a CKiD U25 Bedside eGFR	
<60 mL/min/1.73 m^2^	26.4% (24/91)
**Renal Function at 6 mo Post-transplant**	
Recipient Height (cm)	137.7 ± 2.9 (N = 91)
	[Median = 145.0; Range: 82.0–181.0]
Serum Creatinine (mg/dL)	0.90 ± 0.06 (N = 91)
	[Median = 0.77; Range: 0.22–3.30]
Updated Schwartz Bedside eGFR (mL/min/1.73 m^2^) ^3^	77.4 ± 3.0 (N = 91)
	[Median = 75.9; Range: 19.7–153.9]
CKiD U25 Bedside eGFR (mL/min/1.73 m^2^) ^4^	74.4 ± 2.6 (N = 91)
	[Median = 73.1; Range: 18.3–152.3]
Percentage with an Updated Schwartz Bedside eGFR	
<60 mL/min/1.73 m^2^	27.5% (25/91)
Percentage with a CKiD U25 Bedside eGFR	
<60 mL/min/1.73 m^2^	27.5% (25/91)

^1^ Grades of 1st BPAR were as follows: borderline in 5 cases, IA in 1 case, IB in 1 case, and IIA in 1 case. Antilymphocyte treatment (with rabbit anti-thymocyte globulin) for 1st BPAR was given in 1/8 cases (in the single grade IIA case). ^2^ The 92 study participants were transplanted between 1/13/15 and 12/19/23 (i.e., 2015–2023). The date of last follow-up for this study was 31 December 2024; thus, all 92 pediatric cases were followed for at least 12 mo post-transplant. One patient experienced (death-censored) graft failure during the first 6 mo post-transplant due to renal vein thrombosis (N = 1, at day 1 post-transplant). None of the patients experienced death with a functioning graft during the first 6 mo post-transplant. ^3^ The updated Schwartz bedside formula was used for determining the eGFR here. Of note, no imputation was performed for the single patient who experienced death-censored graft failure at day 1 post-transplant. ^4^ The CKiD U25 bedside formula was used for determining the eGFR here. Of note, no imputation was performed for the single patient who experienced death-censored graft failure at day 1 post-transplant.

**Table 3:** Stepwise linear regression results (multivariable model obtained) for the updated Schwartz bedside log {eGFR} at 1 mo post-transplant (N = 91).

Note: [√] Represents Selection into the Linear Regression Model				
	Univariable	Multivariable Model ^1^		Model Coeff
Variable	*p*-Value	*p*-Value	Coeff ± SE	95% CI
Log {recipient height at 1 mo (cm)}	<0.000001	[√] <0.000001	−0.944 ± 0.173	[−1.288, −0.600]

^1^ The single variable selected into the linear regression model is defined as follows: log {recipient height (cm) at 1 mo post-transplant} (continuous variable). The F-test statistic (with 1 degree of freedom in the numerator and 89 degrees of freedom in the denominator) to include this single variable in the linear regression model was 29.8 (*p* < 0.000001). Once this single variable was selected, no other variables contained additional predictive value (*p* > 0.15). The coefficient of multiple determination, R^2^, achieved for this single-variable linear model was 0.25.

**Table 4:** Stepwise linear regression results (multivariable model obtained) for the updated Schwartz bedside log {eGFR} at 6 mo post-transplant (N = 91).

Note: [√] Represents Selection into the Linear Regression Model				
	Univariable	Multivariable Model ^1^		Model Coeff
Variable	*p*-Value	*p*-Value	Coeff ± SE	95% CI
Log {recipient height at 6 mo (cm)}	0.000003	[√] 0.000003	−0.887 ± 0.178	[−1.241, −0.533]

^1^ The single variable selected into the linear regression model is defined as follows: log {recipient height (cm) at 6 mo post-transplant} (continuous variable). The F-test statistic (with 1 degree of freedom in the numerator and 89 degrees of freedom in the denominator) to include this single variable in the linear regression model was 24.8 (*p* = 0.000003). Once this single variable was selected, no other variables contained additional predictive value (*p* > 0.20). The coefficient of multiple determination, R^2^, achieved for this single-variable linear model was 0.22.

**Table 5:** Stepwise linear regression results (multivariable model obtained) for the CKiD U25 bedside log {eGFR} at 1 mo post-transplant (N = 91).

Note: [√] Represents Selection into the Linear Regression Model				
	Univariable	Multivariable Model ^1^		Model Coeff
Variable	*p*-Value	*p*-Value	Coeff ± SE	95% CI
Log {recipient height at 1 mo (cm)}	0.0009	[√] 0.0009	−0.584 ± 0.170	[−0.921, −0.247]

^1^ The single variable selected into the linear regression model is defined as follows: log {recipient height (cm) at 1 mo post-transplant} (continuous variable). The F-test statistic (with 1 degree of freedom in the numerator and 89 degrees of freedom in the denominator) to include this single variable in the linear regression model was 11.9 (*p* = 0.0009). Once this single variable was selected, no other variables contained additional predictive value (*p* > 0.10). The coefficient of multiple determination, R^2^, achieved for this single-variable linear model was 0.12.

**Table 6:** Stepwise linear regression results (multivariable model obtained) for the CKiD U25 bedside log {eGFR} at 6 mo post-transplant (N = 91).

Note: [√] Represents Selection into the Linear Regression Model				
	Univariable	Multivariable Model ^1^		Model Coeff
Variable	*p*-Value	*p*-Value	Coeff ± SE	95% CI
Log {recipient height at 6 mo (cm)}	0.005	[√] 0.005	−0.502 ± 0.175	[−0.849, −0.155]

^1^ The single variable selected into the linear regression model is defined as follows: log {recipient height (cm) at 6 mo post-transplant} (continuous variable). The F-test statistic (with 1 degree of freedom in the numerator and 89 degrees of freedom in the denominator) to include this single variable in the linear regression model was 8.3 (*p* = 0.005). Once this single variable was selected, no other variables contained additional predictive value (*p* > 0.40). The coefficient of multiple determination, R^2^, achieved for this single-variable linear model was 0.09.

**Table 7:** Stepwise linear regression results (multivariable model obtained) for log {SCr} at 1 mo post-transplant (N = 91).

Note: [√] Represents Selection into the Linear Regression Model				
	Univariable	Multivariable Model ^1^		Model Coeff
Variable	*p*-Value	*p*-Value	Coeff ± SE	95% CI
Log {recipient height at 1 mo (cm)}	<0.000001	[√] <0.000001	1.944 ± 0.173	[1.600, 2.288]

^1^ The single variable selected into the linear regression model is defined as follows: log {recipient height (cm) at 1 mo post-transplant} (continuous variable). The F-test statistic (with 1 degree of freedom in the numerator and 89 degrees of freedom in the denominator) to include this single variable into the linear regression model was 126.2 (*p* < 0.000001). Once this single variable was selected, no other variables contained additional predictive value (*p* > 0.15). The coefficient of multiple determination, R^2^, achieved for this single-variable linear model was 0.59. The F-test of H_0_ when the model coefficient equals 2.0 yields *p* = 0.75; the F-test of H_0_ when the model coefficient equals 1.0 yields *p* < 0.000001.

**Table 8:** Stepwise linear regression results (multivariable model obtained) for log {SCr} at 6 mo post-transplant (N = 91).

Note: [√] Represents Selection into the Linear Regression Model				
	Univariable	Multivariable Model ^1^		Model Coeff
Variable	*p*-Value	*p*-Value	Coeff ± SE	95% CI
Log {recipient height at 6 mo (cm)}	<0.000001	[√] <0.000001	1.887 ± 0.178	[1.533, 2.241]

^1^ The single variable selected into the linear regression model is defined as follows: log {recipient height (cm) at 6 mo post-transplant} (continuous variable). The F-test statistic (with 1 degree of freedom in the numerator and 89 degrees of freedom in the denominator) to include this single variable into the linear regression model was 112.2 (*p* < 0.000001). Once this single variable was selected, no other variables contained additional predictive value (*p* > 0.20). The coefficient of multiple determination, R^2^, achieved for this single-variable linear model was 0.56. The F-test of H_0_ when the model coefficient equals 2.0 yields *p* = 0.53; the F-test of H_0_ when the model coefficient equals 1.0 yields *p* = 0.000003.

## Data Availability

The raw data supporting the conclusions of this article will be made available by the authors on request.

## Abbreviations

BMI	body mass index
BPAR	biopsy-proven acute rejection
C.I.	confidence interval
CKiD	chronic kidney disease in children
cm	centimeters
Coeff	coefficient
d	days
DCGF	death-censored graft failure
DD	deceased donor
DSA	donor-specific antibody
DWFG	death with a functioning graft
eGFR	estimated glomerular filtration rate
KDPI	Kidney Donor Profile Index
LD	living donor
log	natural logarithm
m	meters
mGFR	measured glomerular filtration rate
mo	months
SE	standard error
yr	years
